# The Suppressed Induction of Human Mature Cytotoxic T Lymphocytes Caused by Asbestos Is Not due to Interleukin-2 Insufficiency

**DOI:** 10.1155/2016/7484872

**Published:** 2016-11-15

**Authors:** Naoko Kumagai-Takei, Yasumitsu Nishimura, Hidenori Matsuzaki, Suni Lee, Kei Yoshitome, Hiroaki Hayashi, Takemi Otsuki

**Affiliations:** ^1^Department of Hygiene, Kawasaki Medical School, Kurashiki 701-0192, Japan; ^2^Department of Dermatology, Kawasaki Medical School, Kurashiki 701-0192, Japan

## Abstract

We previously reported that exposure to chrysotile B (CB) asbestos suppressed the induction of mature cytotoxic T lymphocytes (CTLs) during mixed lymphocyte reaction assays (MLRs) with a decrease in the proliferation of immature CTLs. However, the mechanism responsible for the effect of asbestos fibers on the differentiation of CTLs remains unclear. Since interleukin-2 (IL-2) is a regulator of T lymphocyte proliferation, we examined the effect of IL-2 addition on suppressed CTL differentiation in CB-exposed cultures using flow cytometry (FCM). When IL-2 was added at 1 ng/mL on the second day of MLRs, the asbestos-caused decreases in the proliferation and percentages of CD25^+^ and CD45RO^+^ cells in CD8^+^ lymphocytes were not recovered by IL-2 addition, although the decrease in percentage of granzyme B^+^ cells was partially recovered. CD8^+^ lymphocytes from the IL-2-treated culture with asbestos showed the same degree of cytotoxicity as those in cultures without IL-2 or asbestos. These findings indicate that IL-2 insufficiency is not the main cause for the suppressed induction of CTLs by asbestos exposure, although they suggest a potential for the improvement of such suppressed CTL functions. Secretory factors other than IL-2 in addition to membrane-bound stimulatory molecules may play a role in asbestos-caused suppressed CTL differentiation.

## 1. Introduction

The term “asbestos” is derived from the Greek meaning unquenchable and is now utilized as a generic term for a family of naturally occurring fibrous silicate minerals with a crystalline structure [1, 2]. These minerals have been greatly valued for their thermal resistance, flexibility, and durability. Asbestos minerals which consist of a silicate core are subclassified into two groups, amphibole and serpentine, according to the fiber morphology. Amphibole asbestos consists of sharp brittle javelin-shaped fibers with a high length-to-width ratio. This group includes crocidolite, amosite, tremolite, actinolite, and anthophyllite. In contrast, serpentine asbestos, such as chrysotile, comprises long curved fibers [1].

While asbestos possesses beneficial properties, as described above, the association between mesothelioma and asbestos exposure is undisputed [3, 4]. Wagner et al. reported the first association between asbestos exposure and mesothelioma in 1960 [5]. Following Wagner et al.'s study of mesothelioma subsequent to environmental and occupational exposure to asbestos, epidemiological and case-control studies from many industrialized nations have documented rising rates of malignant mesothelioma (MM) following the heavy commercial use of asbestos [3]. Studies have largely focused on the properties of asbestos fibers that are important in the development of MM and the mechanisms of action of asbestos in the multistage carcinogenic process. Asbestos fibers at cytotoxic concentrations cause chromosomal changes, DNA damage, and oxidative DNA lesions in mesothelial cells* in vitro* [6, 7]. The physical and chemical properties of asbestos are influenced by the type and proportion of other metals within the core structure, which may explain the differing carcinogenic potential of various fibers [1].

In fact, the induction of malignant mesothelioma by exposure to asbestos is not a rapid process and takes a long period to develop [8–10]. This suggests the possibility that the development of malignant mesothelioma might be related to other functional alterations, such as those implicated by the idea that exposure to inhaled asbestos might gradually impair the immune response. On the basis of this hypothesis, we have thus far revealed several findings that include alteration in the expression profile of natural killer (NK) cell-activating receptors on human NK cells and functional alterations of CD4^+^ T cells following exposure to asbestos [11, 12].

Recently we reported that asbestos exposure suppressed the differentiation of human mature CTLs during MLRs and was accompanied by decreases in the proliferation of immature CTLs [13].CD8^+^ lymphocytes in culture following exposure to asbestos showed impaired cytotoxicity with decreases in the proliferation and percentages of CD25^+^ and CD45RO^+^ cells in CD8^+^ lymphocytes and an increase in percentage of CD45RA^+^ cells, compared with those in control cultures. Additionally, we reported that patients with mesothelioma showed a decrease in perforin^+^ cell levels in CD8^+^ lymphocytes following stimulation with phorbol 12-myristate 13-acetate and ionomycin, whereas most of the healthy and plaque-positive individuals retained those cell levels following stimulation [14].

In the present study, we focused on investigating the mechanism of the previously reported phenomenon, asbestos-caused suppressed differentiation of mature CTLs with decreased proliferation of immature CTLs. IL-2 is a necessary cytokine for immature CTLs to proliferate during development into mature CTLs [15]. Therefore, we investigated whether IL-2 insufficiency might contribute to the suppressed induction of CTL upon exposure to asbestos. Previously we examined the production of IL-2 during MLRs upon exposure to asbestos [13]. However, after 7 days of the MLRs, the amount of IL-2 was very low in all culture supernatants assayed, and there were no differences in the production of IL-2 between the control culture and the culture with CB asbestos. We hypothesized that IL-2 may play an earlier role in the MLRs. Therefore, in the present study, we examined the production of IL-2 at the early period during MLRs. Additionally, IL-2 was added at 1 ng/mL on the second day of the MLRs to examine the effect of IL-2 addition on suppressed CTL differentiation in cultures exposed to CB asbestos.

## 2. Materials and Methods

### 2.1. MLRs

Peripheral blood mononuclear cells (PBMCs), isolated from heparinized blood by using a Ficoll-Hypaque density gradient (Separate-L, Muto Pure Chemicals Co. Ltd., Tokyo, Japan), were suspended in RPMI 1640 medium (Sigma-Aldrich, St. Louis, MO, USA) supplemented with 10% heat-inactivated fetal bovine serum (Medical and Biological Laboratories Co., Ltd., Nagoya, Japan), 100 *μ*g/mL streptomycin, and 100 U/mL penicillin (Meiji Seika Pharma Co., Ltd., Tokyo, Japan). For the MLRs, 1.5 × 10^5^ PBMCs were cultured with 5.0 × 10^4^ allogenic PBMCs, which had been treated with irradiation of 40 Gy according to a previous method [16], and CB asbestos at 5 *μ*g/mL. Following 2 days of the MLRs, IL-2 (Peprotech, Rocky Hill, NJ, USA) was added at 100 pg/mL or 1 ng/mL for 5 days. International Union Against Cancer (UICC) standard CB was kindly provided by the Department of Occupational Health at the National Institute for Occupational Health of South Africa [17]. All blood samples were taken from healthy volunteers who provided informed consent. The project was approved by the Institutional Ethics Committees of Kawasaki Medical School.

### 2.2. Enzyme-Linked Immunosorbent Assays (ELISA)

After 2, 4, and 7 days of the MLRs, the culture supernatants were collected and assayed for the production of IL-2 using Quantikine ELISA kit (R&D Systems, Inc., Minneapolis, MN, USA). Four independent experiments were performed from three individuals.

### 2.3. Measurement of Cytotoxicity

Cytotoxicity against allogenic target cells was evaluated using FCM as previously described [8]. Allogenic PBMCs were stained using Vybrant™ DiO Cell-Labeling Solution (Molecular Probes, Inc., Eugene, OR, USA) during incubation for 20 mins at 37°C. After DiO-stained cells were washed with phosphate-buffered saline (PBS), effector cells (PBMCs harvested after MLRs) were incubated with 5000 DiO-labeled allogenic PBMCs in 96-well round bottom plates at several different effector/target (E/T) ratios for 5 h at 37°C in 5% CO_2_. Three wells were prepared for each experimental group. Following incubation, cells were collected and stained with propidium iodide (PI) and then analyzed for the percentage of PI^+^ cells among the total DiO-labeled cells (representing the percentage of lysed cells) using FACS Calibur™ (Becton Dickinson, Franklin Lakes, NJ, USA). Two independent experiments were performed. In part of the experiments, CD8^+^ lymphocytes were stained and sorted with phycoerythrin-cychrome 5- (PC5-) conjugated anti-CD8 antibody (Ab) (Beckman Coulter, Inc., Brea, CA, USA) and FACSAria™ (Becton Dickinson) and then purified from the cells harvested after 7 days of the MLRs and assayed for cytotoxicity as described above. Two independent experiments were performed.

### 2.4. Assay for Expression Level of Cell-Surface and Intracellular Molecules

To examine the expression level of molecules on the cell surface, cells harvested after the MLRs were washed with PBS containing 2% FBS and then stained with the following Abs: PC5-conjugated anti-CD8 and fluorescein isothiocyanate- (FITC-) conjugated anti-CD3, FITC-conjugated anti-CD25 (Becton Dickinson), phycoerythrin- (PE-) conjugated anti-CD45RA, or PE-conjugated anti-CD45RO (BioLegend, San Diego, CA, USA) at room temperature in the dark for 30 min. Cells were then washed with PBS containing 2% FBS and resuspended in 0.3 mL of PBS containing 2% FBS for FCM analysis. To examine the expression level of intracellular granzyme B, cells were harvested after the MLRs and surfaces were stained with PC5-conjugated anti-CD8 Ab as described above. Surface stained cells were washed with PBS containing 2% FBS and then fixed with 3.7% formaldehyde for 15 min. Fixed cells were washed with PBS containing 2% FBS. Fixed cells were permeabilized with 0.1% Triton 100 and stained with R-phycoerythrin- (RPE-) conjugated anti-granzyme B Ab (AbD Serotec, Oxford, UK) at room temperature in the dark for 30 min. Cells were then washed and resuspended as described above. The percentage of cells positive for each parameter was analyzed using FCM. Four independent experiments were performed.

### 2.5. Analysis of Granzyme B Production in Proliferating and Nonproliferating CD8^+^ Lymphocytes

To examine the effect of IL-2 on the expression level of intracellular granzyme B in proliferating and nonproliferating CD8^+^ lymphocytes, PBMCs were stained using carboxyfluorescein diacetate succinimidyl ester (CFSE) (Molecular Probes) and then washed and used for MLRs. After MLRs, cells were harvested and stained with PC5-conjugated anti-CD8 and RPE-conjugated anti-granzyme B Abs as described above. The percentage of granzyme B^+^ cells in proliferating CFSE-negative CD8^+^ lymphocytes or nonproliferating CFSE^+^ CD8^+^ cells was analyzed using FCM. Four independent experiments were performed.

### 2.6. Statistical Analysis

Significance of difference (*p* < 0.05) was determined using an analysis of variance with the post hoc test of Student-Newman-Keuls or paired Student's *t*-test.

## 3. Results

### 3.1. Production of IL-2 during MLRs upon Exposure to Chrysotile B Asbestos

**Figure 1:**
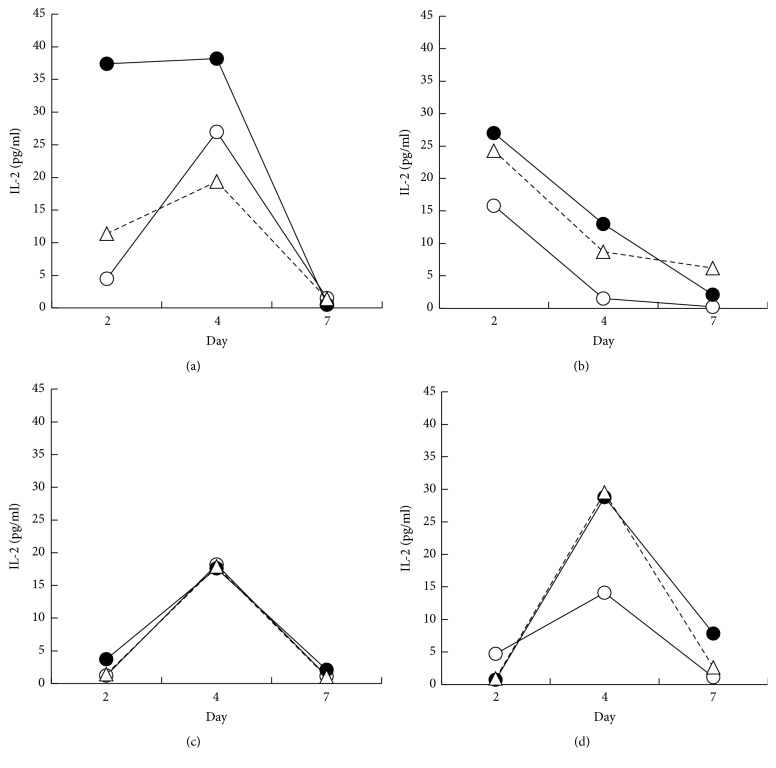
Production of IL-2 during the MLRs upon exposure to asbestos. After 2, 4, and 7 days of the MLRs, culture supernatants were harvested from the three groups, representing no stimulation (open circle), allostimulation (closed circle), and CB-exposed allostimulation (open triangle), and assayed for the production of IL-2 by ELISA. Data (a–d) from one of four independent experiments using PBMCs from three individuals.

To examine the production of IL-2 at days 2, 4, and 7 after the MLRs, the supernatants from cultures of PBMCs stimulated allogenically in the absence or presence of CB asbestos were harvested. For part of the cultures, PBMCs were cultured alone and the supernatants were used as a group without allogenic stimulation. In 3 of the 4 experiments, the production of IL-2 tended to increase following allogenic stimulation to reach a peak at day 4 (Figures 1(a), 1(c), and 1(d)). Some of the CB-exposed cultures showed a decrease in IL-2 compared with the control culture without CB at day 4 (Figures 1(a) and 1(b)). In contrast, in the other CB-exposed cultures, the level of IL-2 production was the same as the control culture (Figures 1(c) and 1(d)). Thus, there were no differences in the production of IL-2 between trials involving the absence or presence of exposure to CB.

### 3.2. Effect of IL-2 on Percentage and Number of CD3^+^CD8^+^ Cells in PBMCs Stimulated upon Exposure to Asbestos

**Figure 2:**
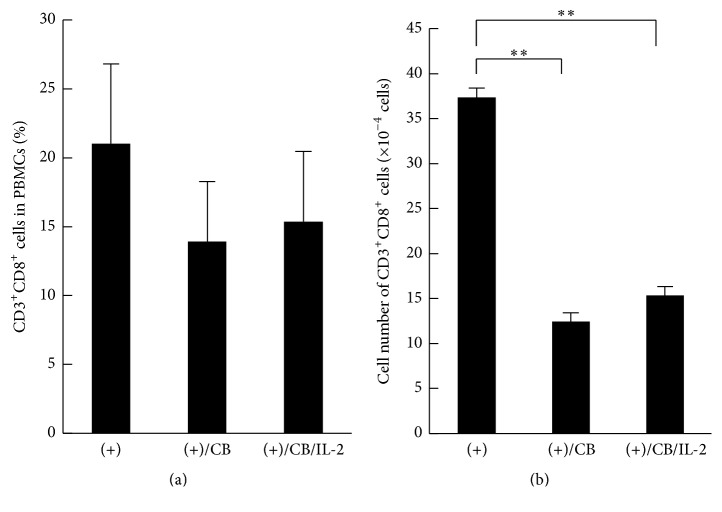
The percentage and number of CD3^+^CD8^+^ cells in PBMCs stimulated with allogenic PBMCs upon exposure to CB with IL-2. Freshly purified PBMCs were cultured with irradiated allogenic stimulator PBMCs in the presence of CB and the absence ((+)/CB) or presence ((+)/CB/IL-2) of IL-2. PBMCs were also cultured with allogenic PBMCs as a control group ((+)) without asbestos and IL-2. The percentage (a) and number (b) of CD3^+^CD8^+^ cells were measured by FCM in PBMCs harvested from 10 wells after culturing. Data represent the mean + SD from four independent experiments using PBMCs. Significant differences are indicated by asterisks (^*∗∗*^ *p* < 0.01). (+), the culture with allogenic PBMCs without CB; (+)/CB, the culture with allogenic PBMCs with CB; (+)/CB/IL-2, the culture with allogenic PBMCs with CB and IL-2.

Although there were no significant differences in the production of IL-2 among the culture groups, the consumption of IL-2 by expanding T lymphocytes would mask the difference in IL-2 production. If insufficient production of IL-2 caused suppressed proliferation of CD3^+^CD8^+^ cells upon exposure to asbestos, exogenous IL-2 should restore such suppressed proliferation. Therefore, IL-2 was added to the asbestos-exposed culture at day 2 after the MLRs, and cells were harvested at day 7 to measure the percentage and number of CD3^+^CD8^+^ cells, since our previous study showed that the number of CFSE-negative cells, proliferating or going to the end of proliferation, increased markedly in CD8^+^ lymphocytes by stimulation with allogenic PBMCs from day 6 to day 7 of the MLRs [13]. There was no statistically significant difference in the percentages of CD3^+^CD8^+^ cells between cultures with and without CB, which was similar to our previous report [13] (Figure 2(a)). The percentage of CD3^+^CD8^+^ cells in the IL-2-treated culture was the same as that present in the CB-exposed culture. It was also reconfirmed that exposure to CB causes a significant decrease in the number of CD3^+^CD8^+^ cells. Furthermore, the addition of IL-2 did not restore the asbestos-caused decrease in the number of CD3^+^CD8^+^ cells (Figure 2(b)). These results indicate that the addition of IL-2 to CB-exposed cultures did not affect either the percentage or the number of CD3^+^CD8^+^ cells.

### 3.3. Effect of IL-2 on Differentiation of Naïve CD8^+^ T Cells into CTLs

**Figure 3:**
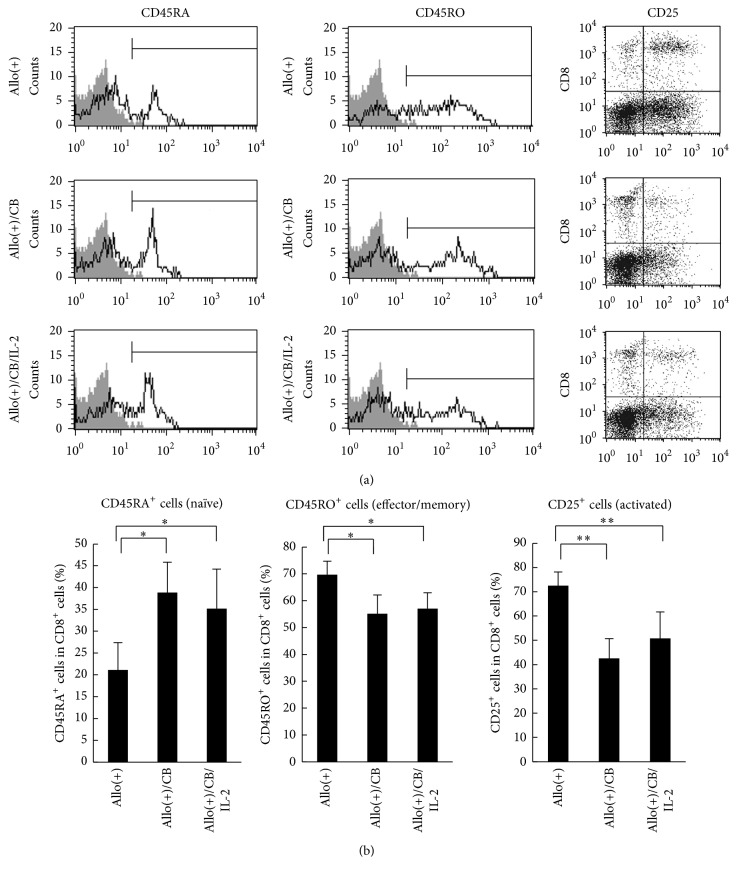
The percentage of cell-surface CD45RA-, CD45RO-, and CD25-positive cells in CD8^+^ lymphocytes stimulated with allogenic cells upon exposure to CB with IL-2. PBMCs were harvested from the three groups, representing allostimulation, CB-exposed allo-stimulation, and CB-exposed allostimulation with IL-2, and assayed for the percentage of cells positive for CD45RA, CD45RO, and CD25 using FCM. (a) Representative histograms of cell-surface CD45RA and CD45RO expressed on CD8^+^ lymphocytes. Representative dot plots of CD25 versus CD8 on PBMCs. (b) Cumulative data showing percentage of CD45RA-, CD45RO-, and CD25-positive cells in CD8^+^ lymphocytes. Data represent the mean + SD from four independent experiments using PBMCs. Significant differences are indicated by asterisks (^*∗*^ *p* < 0.05, ^*∗∗*^ *p* < 0.01). Allo(+), the culture with allogenic PBMCs without CB; allo(+)/CB, the culture with allogenic PBMCs with CB; allo(+)/CB/IL-2, the culture with allogenic PBMCs with CB and IL-2.

To examine the effect of IL-2 addition on asbestos-caused suppressed differentiation of naïve CD8^+^ T cells into effector/memory cells, we analyzed the expression of several cell-surface molecules on CD8^+^ lymphocytes in the culture of PBMCs exposed to CB supplemented with IL-2. The percentage of cells positive for CD45RA and CD45RO, expressed on naïve and effector/memory cells, respectively [18, 19], as well as CD25 cells, expressed on activated cells [20], in CD8^+^ lymphocytes was measured after 7 days of the MLRs, the time point that had also been utilized to confirm the differentiation of effector/memory cells in our previous study [13] (Figure 3(a)). The decrease in CD45RA^+^ naïve cells in CD8^+^ lymphocytes, resulting from stimulation with allogenic PBMCs, was suppressed upon exposure to CB (Figure 3(b)). The increases in CD45RO^+^ effector/memory cells and CD25^+^ activated cells in CD8^+^ lymphocytes were also suppressed by exposure to CB. These results agree with those previously reported in which exposure to CB during MLRs suppressed differentiation into CTLs [13].In the present study, the addition of IL-2 did not restore the suppression of increased levels of CD25^+^ and CD45RO^+^ cells in CD8^+^ lymphocytes or the suppressed decrease in CD45RA^+^ cell levels. These results indicate that exogenous supplementation of IL-2 did not lead to appropriate differentiation of CTLs upon exposure to CB during the MLRs.

### 3.4. Effect of IL-2 on Asbestos-Caused Suppression of Allogenic Cytotoxicity

**Figure 4:**
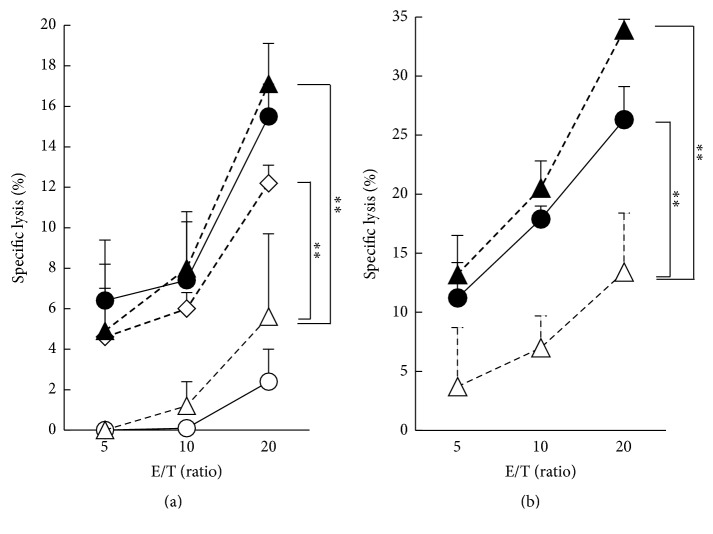
Cytotoxicity of PBMCs or CD8^+^ lymphocytes stimulated with allogenic cells upon exposure to asbestos with IL-2. PBMCs were harvested after the MLRs and assayed for allogenic cytotoxicity using FCM. (a) Dose-dependent allogenic cytotoxicity of cultured PBMCs. PBMCs cultured alone (open circle) or with allogenic PBMCs in the absence (closed circle) or presence of CB (open triangle) or with CB and IL-2 at 100 pg/mL (open diamond) or with CB and IL-2 at 1 ng/mL (closed triangle) as effectors are shown. The percentage of specific lysis induced by effector cells was calculated as follows: (percentage of lysed cells − percentage of spontaneously dead cells)/(100 − percentage of spontaneously dead cells) × 100, where the percentage of spontaneously dead cells represented the percentage of dead cells in target cells harvested from the well without effector cells. Representative data from one of two independent experiments using PBMCs. Data represent the mean + SD from three wells at each E/T ratio. A significant difference is indicated by asterisks (^*∗∗*^ *p* < 0.01). (b) Cytotoxicity of CD8^+^ lymphocytes purified from PBMCs cultured with allogenic cells (closed circles), or with allogenic cells in the presence of CB (open triangles), or with CB and IL-2 at 1 ng/mL (closed triangle). The percentage of specific lysis induced by effector cells was calculated in the same manner as in (a). Representative data from one of two independent experiments using PBMCs. Data represent the mean + SD from three wells at each E/T ratio. A significant difference is indicated by asterisks (^*∗∗*^ *p* < 0.01).

To examine for the effect of IL-2 addition on asbestos-caused suppressed cytotoxicity, we analyzed the cytotoxicity of PBMCs or purified CD8^+^ lymphocytes derived from cultures with allogenic PBMCs and CB supplemented with IL-2.  Figure 4 shows the allogenic cytotoxicity of PBMCs (a) and sorted CD8^+^ lymphocytes (b) in an E/T-ratio-dependent manner. PBMCs cultured with allogenic cells showed obvious cytotoxicity, whereas decreased cytotoxicity was observed in PBMCs exposed to CB during the MLRs. Contrary to our expectation, PBMCs cultured in media supplemented with 100 pg/mL IL-2 exhibited significantly increased allogenic cytotoxicity. Moreover, cells cultured in 1 ng/mL IL-2 showed the same degree of cytotoxicity as those of the allogenic control culture without IL-2 and asbestos (Figure 4(a)). Thus, as shown in Figure 2(b), although exposure to CB regardless of IL-2 treatment resulted in a decreased number of total CD8^+^ T cells in PBMCs, the addition of IL-2 restored the suppressed allogenic cytotoxicity of PBMCs exposed to CB. To remove the difference in the number of total CD8^+^ T cells among the cell groups within PBMCs and to clarify the effect of IL-2 on the allogenic cytotoxicity of CD8^+^ lymphocytes themselves, we purified CD8^+^ lymphocytes from cultured PBMCs and examined the allogenic cytotoxicity of CD8^+^ T cells after the MLRs. CD8^+^ cells purified from PBMCs exposed to CB during the MLRs showed decreased cytotoxicity compared with CD8^+^ cells from PBMCs after the MLRs without CB, which was similar to the results in our previous report [13] (Figure 4(b)). Similar to the results obtained concerning the cytotoxicity of whole PBMCs, CD8^+^ lymphocytes from a 1 ng/mL IL-2-treated culture with asbestos showed the same degree of cytotoxicity as those of the control culture without IL-2 or asbestos (Figure 4(b)). These results indicate that the addition of IL-2 to CB-exposed cultures led to recovery of the cytotoxicity of CD8^+^ lymphocytes for allogenic targets.

### 3.5. Effect of IL-2 on Percentage of Granzyme B-Positive Cells in CD8^+^ Lymphocytes

**Figure 5:**
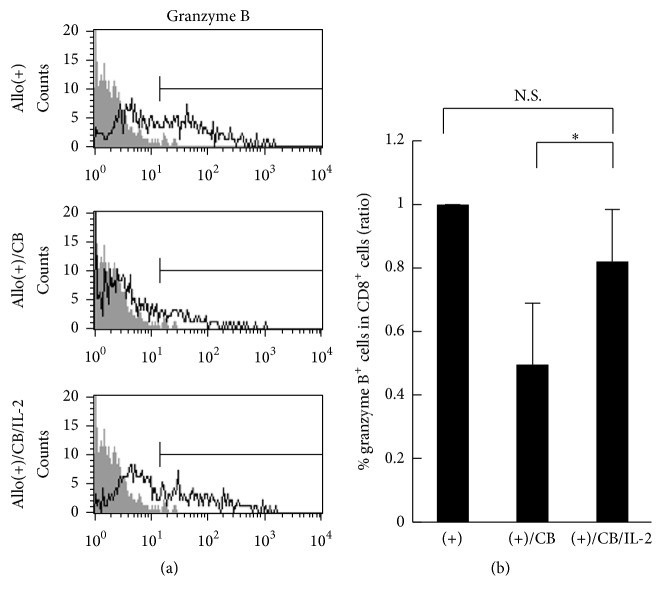
The percentage of granzyme B-positive cells in CD8^+^lymphocytes stimulated with allogenic cells upon exposure to CB with IL-2. PBMCs were harvested from the three groups, representing allostimulation, CB-exposed allostimulation, and CB-exposed allostimulation with IL-2, and assayed for the percentage of cells positive for intracellular granzyme B using FCM. (a) Representative histograms of intracellular granzyme B in CD8^+^lymphocytes. A nonstained control (gray) is shown in each panel. (b) Cumulative data showing the ratio of each group to the allostimulation control was calculated and compared among the groups. Data represent the mean + SD from four independent experiments using PBMCs. Significant differences are indicated by an asterisk (^*∗*^ *p* < 0.05). No significant difference (N.S.) is also indicated. (+), the culture with allogenic PBMCs without CB; (+)/CB, the culture with allogenic PBMCs with CB; (+)/CB/IL-2, the culture with allogenic PBMCs with CB and IL-2.

We analyzed the percentage of CD8^+^ lymphocytes positive for intracellular granzyme B, a representative mediator of target cell death accomplished by CTLs, since our previous study showed that CD8^+^ lymphocytes from PBMCs exposed to CB during the MLRs displayed suppressed cytotoxicity with a decrease in percentages of granzyme B^+^ cells in our previous study [13]. It was again confirmed that the percentage of granzyme B^+^ cells in CD8^+^ lymphocytes increased following allogenic stimulation, whereas the percentage of granzyme B^+^ cells in CD8^+^ lymphocytes was significantly lower following exposure to CB during the MLRs (0.5 ± 0.2 ratio (mean ± S.D.)), which was similar to the results in our previous report [13]. In accordance with the increase in cytotoxicity of CD8^+^ lymphocytes (Figure 4(b)), the addition of IL-2 partially, but not fully, restored the asbestos-caused decrease in the percentage of granzyme B^+^ cells (0.8 ± 0.2 ratio) (Figures 5(a) and 5(b)).

### 3.6. Effect of IL-2 on Induction of Granzyme B in Proliferating or Nonproliferating CD8^+^ Lymphocytes

**Figure 6:**
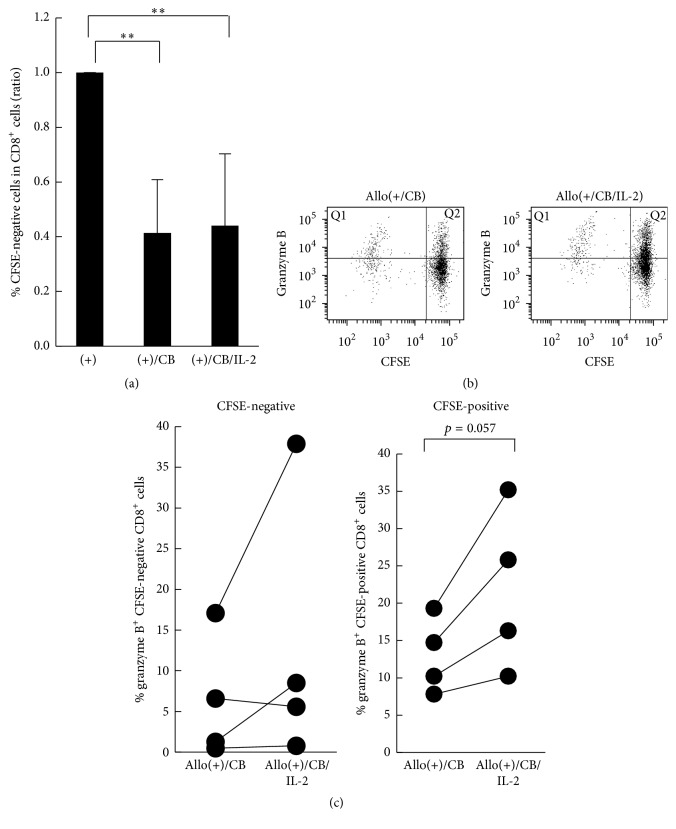
The induction of granzyme B in proliferative and nonproliferative CD8^+^ lymphocytes in the presence of asbestos with IL-2. PBMCs were harvested from the two groups, representing CB-exposed allostimulation and CB-exposed allostimulation with IL-2, and assayed for the percentage of cells positive for granzyme B in CFSE-negative proliferating or CFSE-positive nonproliferating CD8^+^ lymphocytes using FCM. (a) Cumulative data showing the ratio of each group to the allostimulation control was calculated and compared among the groups. Data represent the mean + SD from four independent experiments using PBMCs. Significant differences are indicated by asterisks (^*∗∗*^ *p* < 0.01). (b) Representative dot plots of granzyme B versus CFSE in CD8^+^ lymphocytes. Granzyme B-positive cells of CFSE-negative CD8^+^ lymphocytes (Q1) and granzyme B-positive nondividing cells of CD8^+^ lymphocytes (Q2) were gated for analysis. (c) Cumulative data showing the percentage of granzyme B-positive cells in CFSE-negative or CFSE-positive CD8^+^ lymphocytes. Data represent values from four independent experiments using PBMCs. (+), the culture with allogenic PBMCs without CB; (+)/CB or Allo(+/CB), the culture with allogenic PBMCs with CB; (+)/CB/IL-2 or Allo(+/CB/IL-2), the culture with allogenic PBMCs with CB and IL-2.

As mentioned above, the number of CD8^+^ lymphocytes following exposure to CB did not increase by the addition of IL-2, whereas the cytotoxicity and granzyme B^+^ cell levels increased. Therefore, we set out to determine whether the restored increase in granzyme B^+^ cell levels induced by the addition of IL-2 might be accompanied by a recovery of cell proliferation. PBMCs were stained using CFSE before the MLRs to detect CFSE-negative proliferating cells. After 7 days of the MLRs, PBMCs were collected and stained using granzyme B and CD8 antibodies. As shown in Figure 6(a), addition of IL-2 did not restore the asbestos-caused suppressed proliferation of CD8^+^ lymphocytes during the MLRs. As shown in Figures 6(b) and 6(c), the percentage of CFSE-positive (nonproliferating) and granzyme B-positive cells in CD8^+^ lymphocytes was 35.2% in asbestos-exposed cultures with exogenous IL-2, which tended to be higher than that observed in cultures without IL-2, being 19.3% (*p* = 0.057). In contrast, the level of CFSE-negative (proliferating) and granzyme B-positive cells did not increase with the addition of IL-2 in 3 of the 4 experiments. These results, together with the results mentioned above, indicate that exogenous addition of IL-2 did not result in appropriate CTL differentiation with cell proliferation but improved asbestos-caused suppressed cytotoxicity and partially restored intracellular granzyme B.

## 4. Discussion

Previously, we demonstrated that asbestos exposure suppressed the differentiation of mature CTLs and was accompanied by a decrease in the proliferation of immature CTLs [13]. However, the mechanism responsible for the effect of asbestos fibers on the differentiation of cytotoxic T cells has hitherto remained unknown. In this study, IL-2 addition partially recovered the percentage of granzyme B and the same degree of cytotoxicity as those of the control culture without IL-2 or asbestos, although it did not restore the number of CD3^+^CD8^+^ cells, the proliferation of CD8^+^ lymphocytes, or the percentage of CD45RA^+^, CD45RO^+^, and CD25^+^ cells in CD8^+^ lymphocytes after the MLRs. These findings indicate that IL-2 addition did not restore the asbestos-caused suppressed differentiation and proliferation of CD8^+^ lymphocytes by stimulation with allogenic PBMCs. The present study suggests a potential for improvement of such suppressed CTL functions, whereas it demonstrated that IL-2 insufficiency is not the main cause for the suppressed induction of CTLs with decreased proliferation of CD8^+^ lymphocytes upon exposure to asbestos.

CD8^+^ lymphocytes from IL-2-treated cultures with asbestos showed the same degree of cytotoxicity as those in cultures without IL-2 or asbestos, although IL-2 addition did not restore the suppressed proliferation of CD8^+^ lymphocytes and CTL differentiation upon exposure to asbestos. Therefore, there remains the possibility that the increase in cytotoxicity is not specific against the allogenic target. However, it is known that noncognate antigen or some cytokines such as IL-2 without any antigens can bystander-activate polyclonal memory phenotype cells, but not naïve CD8 cells. Another group also reported that cultures with IL-2, and without antigen, induced the cytotoxic capacity in CD8^+^ T cells by anti-CD3 antibody redirected lysis of Fc IgG-receptor-bearing P815 cells [21]. These findings indicated that bystander-activated CD8^+^ T cells could kill targets if they make contact with target cells. Thus, in this study, polyclonal memory CD8^+^ T cells included in PBMCs before the MLRs might be bystander-activated and kill targets by the addition of IL-2. It is noteworthy that asbestos-exposed CD8^+^ lymphocytes showed a potential to recover cytotoxicity, although this was not accompanied by appropriate proliferation and CTL differentiation. The results of our present study can contribute towards the development of a strategy for the improvement of antitumor immunity in asbestos-exposed patients to avoid the development of malignant mesothelioma.

Thus, the partial recovery of granzyme B expression and enhanced cytotoxicity in CD8^+^ lymphocytes by IL-2 addition indicate that memory CD8^+^ T cells were activated in a TcR-independent manner (bystander activation), as described above. With such bystander activation, some memory CD8^+^ T cells are known to be activated to engage in cell division [21]. However, in our present study, IL-2 addition did not restore the suppressed proliferation of CD8^+^ lymphocytes exposed to asbestos during the MLRs. This question might be explained as follows. Tamang and coworkers reported that CD8^+^ T cells can be partially activated to express granzyme B without being activated to engage in cell division by IL-2 in the absence of antigens [21]. Recently, Arneja et al. reported that the proliferation of CD8^+^ T cells requires continuous Janus kinase (JAK) signal transduction from IL-2 receptor, but not its signal strength [22].In contrast, cell size and metabolic activity increased with increasing cytokine dose (JAK signal strength) independent of time period in culture with IL-2 [22]. Although we have no data to account for the fact that IL-2 addition did not restore asbestos-caused suppressed proliferation of CD8^+^ lymphocytes, it is possible that such incomplete recovery caused by IL-2 addition might be due to a mechanism related to the aforementioned findings.

## 5. Conclusion

Our present investigation demonstrated that the suppressed induction of CTLs upon exposure to asbestos is not attributed to IL-2 insufficiency, whereas addition of IL-2 improved the cytotoxicity of asbestos-exposed CD8^+^ lymphocytes, even though in an incomplete manner. The former motivates us to explore a mechanism for suppressed CTL differentiation upon exposure to asbestos, in which secretory factors other than IL-2 in addition to membrane-bound stimulatory molecules might play a role. This approach could facilitate delineation of asbestos-caused suppressed CTL function. These issues will be examined in future studies.

## References

[1] King J. E., Hasleton P. S., O'Byrne K., Rusch V. (2006). The epidemiology and aetiology of malignant mesotheliom. *Malignant Pleural Mesothelioma*.

[2] Craighead J. E., Gibbs A. R., Pooley F., Craighead J. E., Gibbs A. (2008). Mineralogy of asbestos. *Asbestos and Its Diseases*.

[3] Sporn T. A., Roggli V. L., Roggli V. L., Oury T. D., Sporn T. A. (2004). Mesothelioma. *Pathology of Asbestos-Associated Diseases*.

[4] Mossman B. T., Kamp D. W., Weitzman S. A. (1996). Mechanisms of carcinogenesis and clinical features of asbestos-associated cancers. *Cancer Investigation*.

[5] Wagner J. C., Sleggs C. A., Marchand P. (1960). Diffuse pleural mesothelioma and asbestos exposure in the North Western Cape Province. *British Journal of Industrial Medicine*.

[6] Dušinská M., Collins A., Kazimirova A. (2004). Genotoxic effects of asbestos in humans. *Mutation Research*.

[7] Topinka J., Loli P., Georgiadis P. (2004). Mutagenesis by asbestos in the lung of *λ*-lacI transgenic rats. *Mutation Research/Fundamental and Molecular Mechanisms of Mutagenesis*.

[8] McDonald A. D., McDonald J. C. (1978). Mesothelioma after crocidolite exposure during gas mask manufacture. *Environmental Research*.

[9] Selikoff I. J., Hammond E. C., Seidman H. (1979). Mortality experience of insulation workers in the United States and Canada, 1943–1976. *Annals of the New York Academy of Sciences*.

[10] Selikoff I. J., Hammond E. C., Seidman H. (1980). Latency of asbestos disease among insulation workers in the United States and Canada. *Cancer*.

[11] Nishimura Y., Miura Y., Maeda M. (2009). Impairment in cytotoxicity and expression of NK cell-activating receptors on human NK cells following exposure to asbestos fibers. *International Journal of Immunopathology and Pharmacology*.

[12] Maeda M., Nishimura Y., Hayashi H. (2011). Reduction of CXC chemokine receptor 3 in an *in vitro* model of continuous exposure to asbestos in a human T-cell line, MT-2. *American Journal of Respiratory Cell and Molecular Biology*.

[13] Kumagai-Takei N., Nishimura Y., Maeda M. (2013). Effect of asbestos exposure on differentiation of cytotoxic t lymphocytes in mixed lymphocyte reaction of human peripheral blood mononuclear cells. *American Journal of Respiratory Cell and Molecular Biology*.

[14] Kumagai-Takei N., Nishimura Y., Maeda M. (2014). Functional properties of CD8+ lymphocytes in patients with pleural plaque and malignant mesothelioma. *Journal of Immunology Research*.

[15] Lai Y.-P., Lin C.-C., Liao W.-J., Tang C.-Y., Chen S.-C. (2009). CD4+ T cell-derived IL-2 signals during early priming advances primary CD8+ T cell responses. *PLoS ONE*.

[16] Bluman E. M., Schnier G. S., Avalos B. R. (1996). The c-kit ligand potentiates the allogeneic mixed lymphocyte reaction. *Blood*.

[17] Kohyama N., Shinohara Y., Suzuki Y. (1996). Mineral phases and some reexamined characteristics of the International Union against cancer standard asbestos samples. *American Journal of Industrial Medicine*.

[18] Hamann D., Roos M. T. L., van Lier R. A. W. (1999). Faces and phases of human CD8^+^ T-cell development. *Immunology Today*.

[19] Tomiyama H., Matsuda T., Takiguchi M. (2002). Differentiation of human CD8+ T cells from a memory to memory/effector phenotype. *Journal of Immunology*.

[20] Redmond W. L., Ruby C. E., Weinberg A. D. (2009). The Role of OX40-mediated Co-stimulation in T-cell activation and survival. *Critical Reviews in Immunology*.

[21] Tamang D. L., Redelman D., Alves B. N., Vollger L., Bethley C., Hudig D. (2006). Induction of granzyme B and T cell cytotoxic capacity by IL-2 or IL-15 without antigens: multiclonal responses that are extremely lytic if triggered and short-lived after cytokine withdrawal. *Cytokine*.

[22] Arneja A., Johnson H., Gabrovsek L., Lauffenburger D. A., White F. M. (2014). Qualitatively different T cell phenotypic responses to IL-2 versus IL-15 are unified by identical dependences on receptor signal strength and duration. *The Journal of Immunology*.

